# Dynamic Invariant-Specific Representation Fusion Network for Multimodal Sentiment Analysis

**DOI:** 10.1155/2022/2105593

**Published:** 2022-01-24

**Authors:** Jing He, Haonan Yanga, Changfan Zhang, Hongrun Chen, Yifu Xua

**Affiliations:** College of Electrical and Information Engineering, Hunan University of Technology, Zhuzhou 412007, China

## Abstract

Multimodal sentiment analysis (MSA) aims to infer emotions from linguistic, auditory, and visual sequences. Multimodal information representation method and fusion technology are keys to MSA. However, the problem of difficulty in fully obtaining heterogeneous data interactions in MSA usually exists. To solve these problems, a new framework, namely, dynamic invariant-specific representation fusion network (DISRFN), is put forward in this study. Firstly, in order to effectively utilize redundant information, the joint domain separation representations of all modes are obtained through the improved joint domain separation network. Then, the hierarchical graph fusion net (HGFN) is used for dynamically fusing each representation to obtain the interaction of multimodal data for guidance in the sentiment analysis. Moreover, comparative experiments are performed on popular MSA data sets MOSI and MOSEI, and the research on fusion strategy, loss function ablation, and similarity loss function analysis experiments is designed. The experimental results verify the effectiveness of the DISRFN framework and loss function.

## 1. Introduction

Multimodal sentiment analysis (MSA), as an emerging field of natural language processing (NLP), aims to infer the speaker's emotion by exploring clues in multimodal information [1–3]. Many methods in MSA focus on exploring the complex fusion mechanism to improve the performance of MSA [4–6]. However, these fusion technologies present a bottleneck due to the difficulty in obtaining interaction between heterogeneous modes. The common method to solve this problem is to map the heterogeneous feature to the common subspace in the representation learning process [7]. However, some unique features of each mode are ignored by those methods. These unique features can be used as complementary information between modes. Effective use of this complementary information can help the network improve performance. For this consideration, this paper intends to use supplementary information on the basis of shared representation. And then, a dynamic fusion mechanism is established to fuse the modal features to obtain the interactive information. This study mainly aims to explore a sentiment analysis framework based on multimodal representation learning and the dynamical fusion method.

For multimodal representation learning methods, since multimodal data is usually a sequence with different feature dimensions, long-short memory neural network (LSTM) is a powerful tool to deal with such problems [8]. Therefore, different LSTMs are used to extract features of different modalities in many methods, such as memory fusion network (MFN) [9], graph-memory fusion network (Graph -MFN) [10]. However, a single LSTM is difficult to apply to the feature distribution of each mode at the same time. Therefore, there are studies using different networks to represent different modal information, such as tensor fusion network (TFN) [11], low-rank multimodal fusion net (LMF) [12]. It is worth mentioning that the information between modalities was not used fully before fusion in these methods. The shared features and special features of two data sources are captured by domain separation network (DSN) using adversarial learning and soft orthogonal constraint [13]. And then, these features are used to perform domain adaptive tasks. The combination of shared features and special features can effectively solve the problem that the redundant information between different data sources is not fully utilized. In other words, the DSN is improved and adopted to perform multimodal sentiment analysis tasks in this paper. It is named improved joint domain separation network (improved JDSN).

In this paper, the improved JDSN is adopted to learn the joint representation of modality-invariant and modality-specific of all modes in the common–special subspace. The former aims to map all the modes of discourse to the common subspace to shorten the distance between modes to effectively reduce the extra burden of fusion work. The latter aims to extract special representation from each mode as complementary information. Then, the combination of two representations can fully use the complementary information between modes. In addition, the modal interactions were mostly obtained by feature connection fusion in early work [14]. However, these methods are unable to dynamically adjust the contribution of each mode in the fusion process. Mai et al. assumed that the multimodal fusion process is a hierarchical interactive learning process [15, 16] and designed a ARGF network to solve the problem [15]. The ARGF was comprised of two stages: a joint embedding space learning stage and a hierarchical graph fusion net (HGFN) stage. In the HGFN stage, firstly, the unimodal dynamic layer, bimodal dynamic layer, and trimodal dynamic layer are modelled, and then the outputs of each dynamic layer are connected to obtain the interaction features of each mode. However, the method of joint embedding space learning also has a problem that the redundant information was not fully utilized. Therefore, the improved JDSN and HGFN are combined to optimize the network's ability to capture modal interactions by rationally using redundant information in this paper.

In summary, firstly, the applied DSN in this paper is improved in the aspects of the following: (1) The mode of DSN is extended; (2) The orthogonal constraint loss between special representations of different modes is additionally considered (See Section 3.3.1); (3) Adversarial loss is replaced by a more advanced similarity metric (CMD) (See Section 3.3.2); (4) Invariant and specific representation are jointed at the output of the network (see Section 3.2.3). Then, combining the improved JDSN and HGFN, a new framework (DISRFN) is proposed in this paper to deal with MSA problems. The main contributions are as follows:

1. A multimodal sentiment analysis framework (DISRFN) is proposed in this study. It can perform the fusion of various representations dynamically while emphasizing learning invariant and specific joint representations of various modes.
2. A new loss function is designed, which can improve the effect of semantic fusion clustering whilst assisting the model in learning the target subspace representation effectively.
3. The performance analysis experiments of MSA tasks is designed on the benchmark data sets MOSI and MOSEI. The results confirm the advancement of the DISRFN model and fusion strategy, the effectiveness of the loss function, and the rationality of similarity loss function selection.

The remainder of this paper consists of the following parts. In Section 2, the correlation work is briefly reviewed.  Section 3 introduces the structure of the DISRFN model and the proposed learning method in detail.  Section 4 explains the experimental details, parameter settings, and network component design. The experimental results are analyzed in Section 5.  Section 6 shows the summary and prospects.

## 2. Correlation Work

In multimodal sentiment analysis, the mainstream multimodal learning methods include multimodal fusion representation and multimodal representation learning, which will be discussed in this section.

### 2.1. Multimodal Fusion Representation

In recent years, some complex and efficient fusion representation mechanisms have been gradually proposed. Amir Zadeh et al. put forward TFN to obtain the trimodal fusion representations by using the outer product [11]. On this basis, a low-rank multimodal fusion net (LMF) was proposed. This network performs multimodal data fusion employing a low-rank tensor and obtains better results [12]. Mai et al. proposed a strategy “divide and rule, unite many into one” to transfer local tensor and global fusion, which was extended in multiconnected bidirectional long-short time memory network (Bi-LSTM) [17, 18]. In addition to the tensor fusion method, the recursive fusion method has been developed better. For example, a recursive multilevel fusion network (RMFN) is used for specialized and effective fusion through decomposing the fusion problems into several parts [19]. The more attention-based recursive network (MARN) is used to fuse cyclic memory representations of different modes of long-short term hybrid memory networks (LSTHM) by using a more attention block [20]. Hierarchical polynomial fusion network (HPFN) is used to recursively integrate and transfer the local correlation to the global correlation through multilinear fusion [21]. Moreover, the multiview learning method plays an important role in multimodal fusion [22]. For example, MFN designed by Amir Zadeh et al. is used to fuse the memory of different modes of LSTM system based on incremental attention memory network (DAMN) and gated memory network (MVGN) [9], and it is successfully used to solve multiview problems. Furthermore, to analyze the explainability of MFN, the dynamic fusion graph model (DFG) is embedded into MFN, and a Graph-MFN obtained finally has excellent performance and is explainable [10]. Recently, word-level fusion representation has also been a wide concern [23]. For example, a repeated participation variation network (RAVEN) is used to model multimodal language through work representation transfer based on facial expression [24]. Chen et al. modeled the time-dependent multimodal dynamics through cross-modal work alignment [25]. However, most of these methods use complex fusion mechanisms or add additional fusion modules, which will increase the amount of calculation and slow down the speed of network convergence. In contrast, this paper uses a hierarchical mechanism to model the dynamics of each fusion layer, which can quickly fuse the information of each mode.

### 2.2. Multimodal Representation Learning

Multimodal representation learning is mainly divided into two types, namely, common subspace representations and factorised representations. The two types of study on common subspace representations amongst modes are the correlation-based model and adversarial learning-based model. In terms of a correlation-based model, Shu et al. proposed an extensible multilabel canonical correlation analysis (sml-CCA) for cross-modal retrieval [26]. Kaloga et al. proposed a multiview graph canonical correlation analysis based on variational graph neutral network for classification and clustering tasks [27]. Verma et al. proposed a deep network with high-order information and single sequence information (Deep-HOSeq) for fusing multimodal sentiment data [28]. Mai et al. learned the embedding space within invariant mode based on a new encoding-decoding classifier framework in confrontation [15]. Pham et al. proposed a robust joint representation method to learn by shifting between modes under the constraints of cyclic consistency loss [29]. In terms of the adversarial learning-based model, Wu and Qiang et al. proposed the generative adversarial net based on specific mode and sharing and the adversarial hashing algorithm based on deep semantic similarity, respectively, to obtain cross-modal invariance [30, 31]. However, these methods only learn about the shared representation of the model and lack the consideration of the special representation of the modal. For factorized representations, Amir Zadeh et al. proposed a multimodal factorized model (MFM) to factorize multimodal representations into multimodal discriminant factor and multimodal special generation factor [32]. Liang et al. proposed a multimodal baseline model (MMB) to learn the cases of multimodal embedding based on the factorized method [33]. Wang et al. proposed a joint and separate matrix factorized hashing method, which could be used to learn common and specific attributes of multimodal data at the same time [34]. Fang et al. proposed a new semantic enhanced discrete matrix factorized hashing (SDMFN), which could directly extract the common hashing representation from the reconstructed semantic polynomial similar graph, causing the hash code to be more discriminative [35]. Caicedo et al. proposed a multimodal image representation based on nonnegative matrix factorisation to synthesise visual features and text features [36]. However, most of these factorized methods adopt the form of matrix decomposition, which may have the problem of incomplete feature representation. In contrast, the improved JDSN designed in this paper can obtain a richer shared-special representation of each mode in a simpler way.

## 3. The Proposed Method

### 3.1. Task Setting

**Figure 1:**
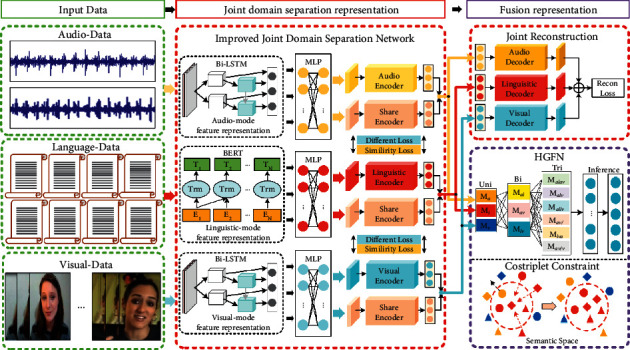
The framework of DISRFN. Note: Bi-LSTM: bidirectional short and long memory network; BERT: bidirectional encoder representation from transformers; MLP: multilayer perception; audio encoder (decoder): encoder (decoder) of auditory mode; linguistic encoder (decoder): encoder (decoder) of linguistic mode; visual encoder (decoder): encoder (decoder) of visual mode; share encoder: shared encoder of three modes; HGFN: hierarchical graph fusion net.

In general, the proposed framework is mainly used to study the trimodal data.  Figure 1 shows the flowchart of the proposed multimodal fusion framework. This framework consists of two parts, as follows: (1) improved JDSN for learning trimodal data-specific shared subspace joint representation; (2) HGFN for fusing trimodal joint representation, thereby realizing dynamical effective semantic clustering. This study introduces this network framework in the following section.

Moreover, the discourse data are divided into N sequences composed of segment S to facilitate detecting emotion in video by using multimodal data. Each segment S includes three low-level feature sequences in linguistic (l), visual (v), and auditory (a) modes. These feature sequences are represented as S_*l*_ ∈ *ℝ*^*t*_*l*_×*d*_*l*_^, S_*v*_ ∈ *ℝ*^*t*_*v*_×*d*_*v*_^, S_*a*_ ∈ *ℝ*^*t*_*a*_×*d*_*a*_^. Amongst them, *t*_m_ and *d*_m_ (*m*∈{l, *v*, *a*}) represent the length of discourse and the dimension of the corresponding feature, respectively. Given this data sequence, the study aims to predict the emotional state of the predefined set. This emotional state is a continuous dense variable *y* ∈ *ℝ*. In addition, to effectively use multimodal data, linguistic (l), visual (v), and auditory (a) trimodal feature sequences, they should be aligned with emotional state label *y*.

The framework of DISRFN is shown in Figure 1: (1) The data of the three modes are fed into the corresponding Bi-LSTM and BERT models to obtain the discourse-level feature representations; (2) The discourse-level feature representations of each mode are fed into the corresponding MLP to obtain the representation of unified dimension; (3) The unified representations of each mode are fed into the corresponding encoder and shared encoder to obtain the shared representations and special representations; (4) The shared representations are added with a special representation of each modal to obtain the joint domain separation representations; (5) The joint domain separation representations of each mode are fed into the corresponding decoder to obtain the reconstruction loss; (6) The joint domain separation representations of each mode are fed into HGFN for dynamic fusion to perform MSA task.

### 3.2. Dynamic Invariant-Specific Representation Fusion Network

#### 3.2.1. Discourse-Level Feature Representation

Firstly, the stacking bidirectional long-short time memory neural network (sLSTM) is used to map the feature sequence (*S*_v_, *S*_a_) in visual (*v*) and auditory (*a*) modes to obtain the underlying features of the sequence. Its output includes the hidden representations of LSTM end state, namely, *F*_v_ and *F*_a_, as follows:

(1) $$\begin{array}{c} F_{v}=s\text{LSTM}\left(S_{v};\theta_{v}^{\text{LSTM}}\right),\\ F_{a}=s\text{LSTM}\left(S_{a};\theta_{a}^{\text{LSTM}}\right), \end{array}$$

where *θ*_*v*_^LSTM^ and *θ*_*a*_^LSTM^ refer to the parameters of sLSTM on visual and auditory modes.

Secondly, for the text feature sequence (*S*_l_) in linguistic mode, most linguistic features are embedded through Glove [37]. However, in recent studies [38], such as the advanced ICCN [39] model, the pretraining BERT model is used as the feature extractor of text discourse. A better result than the Glove method is obtained. Therefore, the feature representation *F*_l_ of text is obtained through the pretraining BERT model, as follows:

(2) $$\begin{array}{c} F_{l}=\text{BERT}\left(S_{l};\theta_{l}^{\text{BERT}}\right), \end{array}$$

where *θ*_*l*_^BERT^ refers to the parameter of the BERT model.

#### 3.2.2. Unified Representation of Features

The dimensions of discourse-level features are different. In order to facilitate the encoding-decoding operation in the back-end network, multilayer perceptron (MLP) is used to unify mapping these features to *O*_m_, as follows:

(3) $$\begin{array}{c} O_{m}=\text{MLP}\left(F_{m};\theta_{m}^{MLP}\right),\left(m\in \left\{l,v,a\right\}\right), \end{array}$$

where *θ*_*m*_^MLP^ refers to a parameter of multilayer perceptron networks in different modes; MLP consists of dense connection layers and a normalized layer activated by relu function.

#### 3.2.3. Improved Joint Domain Separation Representation

In this part, based on the improved JDSN, the unified mapping representation of each mode is factorized into two parts, namely, modality-invariance and modality-specificity. Amongst them, the sharing encoder *E*^*c*^ is used to learn invariant representation in the common subspace to narrow the gap in the heterogeneity between modes [40]. The specific encoder *E*_*m*_^*p*^ is used to capture the specific representation in a specific subspace. The process is as follows.

Firstly, after obtaining the unified mapping vector *O*_m_ of each mode, the mode-sharing encoder *E*^*c*^ (weight sharing) is used to obtain modality-invariant representation (*h*_*m*_^*c*^), and the mode-specific encoder *E*_*m*_^*p*^ is used to extract modality-specific representation (*h*_*m*_^*p*^), as follows:

(4) $$\begin{array}{c} h_{m}^{c}=E^{c}\left(O_{m};\theta^{c}\right),h_{m}^{p}=E_{m}^{p}\left(O_{m};\theta_{m}^{p}\right),\left(m\in \left\{l,v,a\right\}\right), \end{array}$$

where *θ*^*c*^ refers to a parameter of mode-sharing encoder; *θ*_*m*_^*p*^ refers to a parameter of mode-specific encoder; *E*^*c*^ has the same structure as that of *E*_*m*_^*p*^, which is composed of a dense connection layer activated by sigmoid function.

Then, hidden layer vectors *h*_*m*_^*p*^ and *h*_*m*_^*c*^ are generated through feedforward propagation of neural network, and the joint domain separation representation is obtained through vector addition “+”, as follows:

(5) $$\begin{array}{c} h_{m}=h_{m}^{c}+h_{m}^{p},\left(m\in \left\{l,v,a\right\}\right), \end{array}$$

where *h*_*m*_ refers to the joint domain separation representation of mode *m*, and it has the feature representation of shared subspace and specific subspace characteristics.

#### 3.2.4. Hierarchical Graph Fusion Representation

After obtaining the joint domain separation representation of each mode, it is necessary to fuse each representation to obtain the interaction information of each mode.

**Figure 2:**
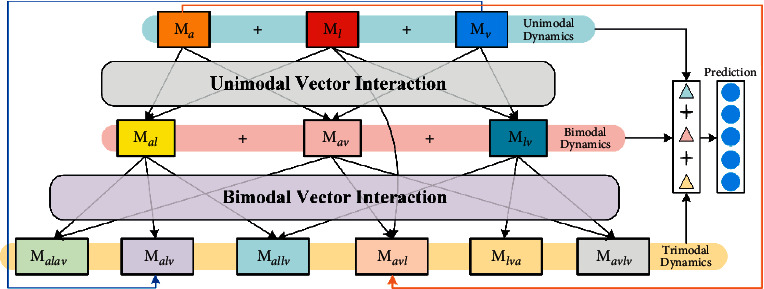
The framework of HGFN.

As shown in Figure 2, HGFN is composed of three dynamic layers (unimodal dynamic layer, bimodal dynamic layer, and trimodal dynamic layer). Unimodal dynamic layer is modeled by self-attention weighting each unimodal information vector. Bimodal dynamic layer is modeled by weighting bimodal information vectors (e.g., M_al_) using the correlation weight between unimodal vectors. Trimodal dynamic layer is constructed through weighting trimodal information vectors (e.g., M_alv_ or M_allv_) by the correlation weight between unimodal vectors. Finally, three dynamic layers are used for vector connection and fusion to realize the dynamic fusion of multimodal features in HGFN. This hierarchical modeling method is more conducive to exploring the interaction between modes [12]. Therefore, HGFN, which can preserve all modal interactions, is introduced to fuse the obtained joint domain separation representations of different modes to explore multimodal interaction in this section. The fusion representation is as follows:

(6) $$\begin{array}{c} \text{Fusion}=\text{HGFN}\left(h_{l},h_{v},h_{a};\theta^{\text{HGFN}}\right), \end{array}$$

where “Fusion” refers to the output of HGFN; *θ*^HGFN^ refers to the parameters of HGFN. Then, the predictive neural network (P) is used for prediction, as follows:

(7) $$\begin{array}{c} \mathrm{Pred}=P\left(\text{Fusion};\theta^{\mathrm{Pr}\text{e}}\right), \end{array}$$

where “Pred” refers to the output of the predictive network; “P” refers to a predictive network, including a standardized layer and the fully connected layers; *θ*^Pre^ refers to the parameter of the predictive network. Moreover, the specific parameters of the model are described in the experimental section.

### 3.3. Learning Process

A joint loss function is newly set to effectively learn the network model, as follows:

(8) $$\begin{array}{c} L_{\text{total}}=L_{\text{task}}+\alpha L_{\text{diff}}+\beta L_{\text{sim}}+\gamma L_{\text{recon}}+\eta L_{\text{trip}}, \end{array}$$

where *α*, *β*, *γ*, and *η* refer to weights of the interaction. They determine the contributions of each loss L_diff_, L_sim_, L_recon,_ and L_trip_ to total loss L_total_. In addition, each loss is analyzed and introduced in the remaining section.

#### 3.3.1. Differential Loss

Some studies have shown that a nonredundant effect can be achieved by applying soft orthogonality constraint to two representation vectors [13, 41]. Therefore, the constraint is used to drive the sharing-encoder E^*c*^ and specific-encoder E_*m*_^*p*^ to perform encoding representation to different aspects, that is, modality-invariant and modality-specific representations. Soft orthogonality constraint is defined as follows.

When training a batch of data, *H*_*m*_^*c*^ and *H*_*m*_^*p*^ are set as the two matrices, respectively. The rows of the two matrices correspond to invariant representation *h*_*m*_^*c*^ and specific representation *h*_*m*_^*p*^ of mode *m* in each batch of data, respectively. The orthogonality constraint of the modal vector is calculated as follows [13]:

(9) $$\begin{array}{c} L_{\text{diff}}=\underset{m\in \left\{l,v,a\right\}}{\sum }{\left\|H_{m}^{cΤ}H_{m}^{p}\right\|}_{F}^{2}+\underset{\begin{array}{c} \left(m_{1},m_{2}\right)\in \left\{\left(l,a\right)\right.,\\ \left(l,v\right),\left.\left(a,v\right)\right\} \end{array}}{\sum }{\left\|H_{m_{1}}^{pΤ}H_{m_{2}}^{p}\right\|}_{F}^{2}, \end{array}$$

where *||* · *||*_*F*_^2^ refers to squared Frobenius norm.

#### 3.3.2. Similarity Loss

Similarity loss (L_sim_) used to constrain shared subspace can reduce the difference in the heterogeneity between the shared representations of different modes [42]. Central moment discrepancy (CMD) is used to measure the difference between two distributions by matching order-wise moment differences of two representations [43]. Compared with other methods (e.g., MMD and DANN), it is a more efficient and concise distance measurement. Therefore, CMD is selected as the similarity loss in this paper. It is defined as follows.

*X* and *Y* are set as bounded random samples with probability distributions *p* and *q* in a compact interval [*a*, *b*]^*N*^, respectively. CMD is defined as follows [43]:

(10) $$\begin{array}{c} \text{CMD}\left(X,Y\right)=\frac{1}{\left|b-a\right|}{\left\|E\left(X\right)-E\left(Y\right)\right\|}_{2}+\sum_{k=2}^{K}\frac{1}{{\left|b-a\right|}^{k}}{\left\|C_{k}\left(X\right)-C_{k}\left(Y\right)\right\|}_{2}\\ C_{k}\left(X\right)=E\left({\left(x-E\left(X\right)\right)}^{k}\right)\\ E\left(X\right)=\frac{1}{\left|X\right|}\sum_{x\in X}x, \end{array}$$

where *E*(*X*) refers to the empirical expectation vector of sample *X*; *C*_*k*_(*X*) refers to the vector of all k-order sample centre moments in the *X* coordinate.

In this paper, the similarity loss is calculated by summing the CMD distances of the shared representations of every two modes. Its representation is as follows:

(11) $$\begin{array}{c} L_{\text{sim}}=\underset{\begin{array}{c} \left(m_{1},m_{2}\right)\in \left\{\left(l,a\right),\right.\\ \left.\left(l,v\right),\left(a,v\right)\right\} \end{array}}{\sum }\text{CMD}\left(h_{m_{1}}^{c},h_{m_{2}}^{c}\right), \end{array}$$

Moreover, the reason for selecting CMD as the similarity loss will be discussed in Experimental part 5.4.

#### 3.3.3. Reconstruction Loss

When soft orthogonality constraint is enforced, the risk of specific encoder learning trivial representation exists. However, the reconstruction loss can be added to ensure that the encoder can capture the details of each mode to solve these problems [13]. Initially, the modal decoder *D*_*m*_ is used to reconstruct the joint domain separation representation vector *h*_*m*_ of mode *m*, and the output of reconstruction is ${\hat{h}}_{m}$. Then, the reconstruction loss is represented by the mean square error loss between *h*_*m*_ and ${\hat{h}}_{m}$, as follows [13]:

(12) $$\begin{array}{c} L_{\text{recon}}=\frac{1}{3}\left(\sum_{m\in \left\{l,v,a\right\}}{\left\|h_{m}-{\hat{h}}_{m}\right\|}_{2}^{2}\right), \end{array}$$

where *||* · *||*_2_^2^ refers to squared L_2_-norm.

#### 3.3.4. Cosine Triplet-Margin Loss

In the fusion representation of joint domain separation representation vector, to ensure the high-level relationship of the similarity between all projects, the representation distance of discourse segments with similar semantics between different modes is minimized through cosine triplet-margin loss L_trip_, and the distance between different discourse segments is maximized [44].

For example, in linguistic and visual modes, a triple representation (*h*_*l*_, *h*_*v*_^+^, *h*_*v*_^−^) is established. Amongst them, visual representation *h*_*v*_^+^ is positively correlated with linguistic representation *h*_*l*_ in semantics. At the same time, visual representation *h*_*v*_^−^ is the contrary. Therefore, the cosine triplet-margin loss of linguistic mode is shown as follows [44]:

(13) $$\begin{array}{c} L_{\text{trip}}^{l}=\sum_{m\in \left\{v,a\right\}}\max\left(\cos\left(h_{l},h_{m}^{-}\right)-\cos\left(h_{l},h_{m}^{+}\right)+\text{margin},0\right), \end{array}$$

where *h*_*m*_^+^, *h*_*m*_^−^ refers to the joint domain separation representation vector of mode *m*; “margin = 1” is a boundary parameter.

In the same way, the cosine triplet-margin loss of visual mode and auditory mode can be described as follows:

(14) $$\begin{array}{c} L_{\text{trip}}^{v}=\sum_{m\in \left\{l,a\right\}}\max\left(\cos\left(h_{v},h_{m}^{-}\right)-\cos\left(h_{v},h_{m}^{+}\right)+\text{margin},0\right), \end{array}$$

(15) $$\begin{array}{c} L_{\text{trip}}^{a}=\sum_{m\in \left\{l,a\right\}}\max\left(\cos\left(h_{v},h_{m}^{-}\right)-\cos\left(h_{v},h_{m}^{+}\right)+\text{margin},0\right). \end{array}$$

Based on formulas (13)–(15), the total cosine triple margin loss is represented as follows:

(16) $$\begin{array}{c} L_{\text{trip}}=L_{\text{trip}}^{l}+L_{\text{trip}}^{v}+L_{\text{trip}}^{a}. \end{array}$$

#### 3.3.5. Task Loss

The mean square error (MSE) is used as the task loss of the network to predict continuous dense variables. For *N*_*b*_ discourse data in one batch, this loss calculation is as follows:

(17) $$\begin{array}{c} L_{\text{task}}=\frac{1}{N_{b}}\sum_{i=0}^{N_{b}}{\left\|y_{i}-{\hat{y}}_{i}\right\|}_{2}^{2}. \end{array}$$

where *y*_*i*_ refers to the actual emotional label; ${\hat{y}}_{i}$ refers to the predictive value of the network.

## 4. Experiment

In this section, the required data sets, evaluation index, and experimental details (experimental environment, experimental parameters, and network structure) are described.

### 4.1. Datasets

The data set is introduced in this section. This data set includes two parts, namely, CMU-MOSI and CMU-MOSEI.

CMU-MOSI data set: this data set is a collection of monologues on YouTube, including videos with 93 comments from different speakers. These common videos consist of 2199 subjective discourses. These discourses are manually marked with continuous opinion scores in the range of −3 to 3. Amongst them, −3/+3 represents strong negative/positive emotions. A total of 1283 segment samples are used for training, 229 segments are used for verification, and 686 segments are used for testing.

CMU-MOSEI data set: it is an improved version of MOSI; it includes 23453 annotated discourse segments, which are from 5000 videos, 1000 different speakers, and 250 different topics. A total of 1283 segment samples are still used for training, 229 segments are used for verification, and 686 segments are used for testing.

The problems on multimodal signal (linguistic, visual, and auditory) acquisition and modal data pretreatment are solved based on CMU-Multimodal SDK^1^ in many studies [45]. This tool library is a machine learning platform used for developing high-level multimodal models and acquiring and processing multimodal data by Amir Zadeh et al. It integrates the acquisition and alignment method of benchmark data sets (MOSI and MOSEI). Similarly, this tool library is used to solve the problems of data acquisition and alignment.

### 4.2. Evaluation Index

This experiment is a regressive task. Therefore, the mean absolute error (MAE) and Pearson correlation coefficient (Corr) are adopted to measure the test results. In addition, the classification index is considered in the experiment, including five-classification accuracy (Acc-5) in affection domain (−2,2), two-classification accuracy (Acc-2) including positive and negative emotion (p/g), and F1score (F1-Score).

### 4.3. Experimental Settings

This method is tested on Pytorch in this section. The grid searching of hyperparameter is performed in a data verification set to identify appropriate hyperparameter, and the best model and hyperparameter are saved. In grid searching, limited option sets for setting hyperparameters are as follows: *α*∈{0.3, 0.4}, *β*∈{0.7, 0.8, 0.9, 1.0}, *γ*∈{0.1, 0.2, 0.3, 0.4, 0.5}, *η*∈{0.01, 0.1} and drop∈{0, 0.1, 0.2, 0.3, 0.4}; the hidden layer sizes of the representation and predictive network can be reviewed from the following: Hid∈{128, 256}, P_h∈{50, 64}.

**Figure 3:**
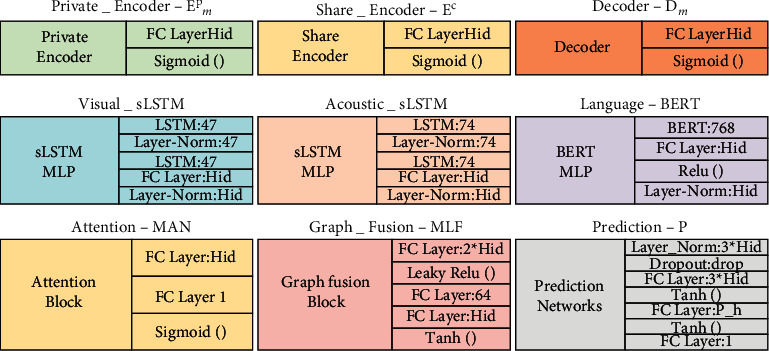
The parameter setting of modules.

In the iterative optimization process, Adam optimizer with max epoch *=* 20, batch_size *=* 16, and learning rate of 0.0001 are used to train the network. The grid searching results of all data sets are shown in Table 1, and based on the hyperparameter settings, Figure 3 shows the model component structural diagram. Note: (1) FC Layer is the dimension of the fully connected layer; (2) LSTM is the dimension of the LSTM hidden layer; (3) Layer-Norm is a dimension of the batch normalization layer; (4) Dropout is the rate of dropout; (5) BERT is the output dimension of the BERT model; (6) Hid/drop/P_h is hyperparameters.

### 4.4. Experimental Process

This section mainly introduces the experimental process, the specific experimental steps are as follows:

1. Manual feature extraction of video and audio: for CMU-MOSI and CMU-MOSEI, Facet^2^ and COVAREP [46] are used to extract the manual features of visual and auditory sequences. Amongst them, the dimensions *d*_*v*_ of the visual feature are 47 and 35, respectively, and the dimension *d*_*a*_ of the auditory feature is 74.
2. Discourse-level feature extraction: for linguistic mode, because the BERT model has text embedding and representation functions, the pretraining model of BERT is directly used to extract linguistic features. Its discourse-level feature is represented as feature representation F_*l*_ with dimension of 768 [47]. And then, visual and auditory features at the discourse-level F_*v*_ and F_*a*_ are obtained based on sLSTM.
3. Unified representation mapping: MLP is adopted to map linguistic, visual, and auditory representation vectors F_*l*_, F_*v*_, and F_*a*_ to an output *O*_m_ with the unified dimension size.
4. Improved joint domain separation representation: *O*_*m*_ is input to sharing encoder and specific encoder to obtain hidden layer representation h_*m*_^*c*^, h_*m*_^*p*^. And then, an improved joint domain separation representation h_*m*_ is obtained through vector addition (h_*m*_^*p*^+h_*m*_^*c*^).
5. Fusion inference: the joint domain separation representation vector is sent to the HGFN to perform fusion and prediction tasks.
6. Calculating loss function and training: loss function is calculated to train the neural network and make cyclic iteration.

## 5. Results and Analysis

Model comparison experiments, research on fusion strategy, research on loss function ablation, and research on similarity loss selection are designed in this section. All experiments are discussed by combining visualization and quantitative analysis.

### 5.1. Model Comparison Experiments Result

In the comparison experiment, some classical models (TFN, LMF, MFN, Gragh-MFN, MARM, and MISA) are reproduced. In addition, some derived fusion model based on LSTHM [17] is designed to comparison with the proposed framework (DISRFN). The result is shown in Tables 2 and 3.

Tables 2 and 3 show that our method achieves the best performance under two data sets. That is, it exceeds the comparison model in terms of MAE, Corr, Acc, and other comprehensive indexes. These results show that the proposed model exceeds some complex fusion mechanisms (e.g., TFN, MFN, and Gragh-MFN) in the performance. The reason is that these methods ignore the exploration of modal invariant space while the proposed method obtains a joint representation of invariant-specific space.

Moreover, it can be seen from the “CPU Clock” items in Tables 2 and 3. Compared with the model that also applies mechanism fusion (TFN, LMF, MFN, Gragh-MFN, MARM, ARGF, LSTHM-DFG, LSTHM-Out Product), the proposed method is at a disadvantage in the aspect of real-time due to the relatively large number of parameters in the representation learning. However, compared with the model that uses additional networks in the fusion part (MISA, LSTHM-AttFusion, LSTHM -Concat), the proposed method has an advantage when it comes to real-time. Therefore, compared with the baseline model, the proposed method has moderate real-time performance when the various MSA indicators are optimal.

In Section 3.2.1, the reason for using the BERT pretraining model to extract discourse-level features of language modality instead of Glove method is explored. Tables 2 and 3 show that, compared with the baseline model based on the Glove word embedding method, and LSTHM*-*derived fusion model, various evaluation indexes are improved significantly by the model using BERT (DISRFN and MISA). It proves that the application of the BERT method is reasonable. Moreover, compared with the MISA model using BERT, the proposed model still has a slight advantage. The difference is probably caused by different fusion strategies. The comparative experiment is carried out in the next section to further discuss the effectiveness of the fusion strategy of this model.

### 5.2. Fusion Strategy Comparison Result

In this section, a fusion strategy comparison experiment is designed in the MOSI data set to verify the effectiveness of the HGFN fusion strategy. The improved JDSN component remains unchanged in the experiment, and the fusion component is replaced with Multi-Attention Fusion (AttFusion), vector concatenation fusion (Concate), dynamic fusion net (DFN), and other strategies. Then, the results are concluded, as shown in Tabel 4.

The results shown in Tabel 4 indicate that HGFN has a significantly improved performance compared with other fusion methods. The reason for these results is that HGFN not only models single-modal, bimodal, and trimodal layers dynamically but also obtains trimodal fusion representations more comprehensively by the splicing mode of various modal layers. Moreover, to verify the dynamicity of the graph fusion network, the weight change of the fusion process is visualized as follows.

**Figure 4:**
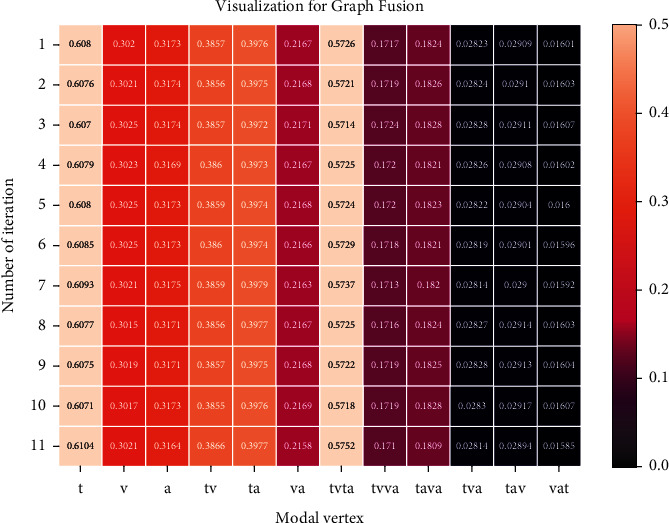
Visualization for Graph Fusion in MOSI sentiment analysis task.

As shown in Figure 4, the vertical axis represents the iteration order, and the horizontal axis represents the interaction information vector in the dynamic layer. The value in the figure represents the weight of the corresponding information vector. The results of vertical axis analysis indicate that the contributions of different discourse segments to the same modal interaction information vector are almost unchanged. The reason is that the modal data are affected by the similarity constraint in the domain separation representation learning prior to fusion, which reduces the fluctuation in the difference amongst all sample representations. Through the observation of the horizontal axis, for single-modal vector weight (the first three columns), the contributions of linguistic mode to the prediction result are the most evident. The reason is that language text is usually the most important information in MSA. For bimodal vector weight (fourth–sixth column), weight “tv” is closer to “ta” and significantly greater than weight “va”. The reason may be that linguistic mode plays a more important role in bimodal fusion than other modes. Through observation of the trimodal vector weight (the seventh–twelfth column), the vector weight obtained by fusing one bimodal vector and one single-modal vector is close to 0. However, the vector weight obtained by fusing two bimodal vectors is dominant in the trimodal information. It indicates that modeling the interaction process of every two bimodal vectors is necessary. And it is also verified that the fusion network can dynamically fuse the multimodal data.

### 5.3. Ablation Study

The loss functions of various components discussed in Section 3.3 play an important role in the implementation of an improved joint domain separation network in Section 3.2. Therefore, the loss function is analyzed and discussed, and visualised and quantitative analysis is conducted based on ablation study.

#### 5.3.1. Visual Presentation

**Figure 5:**
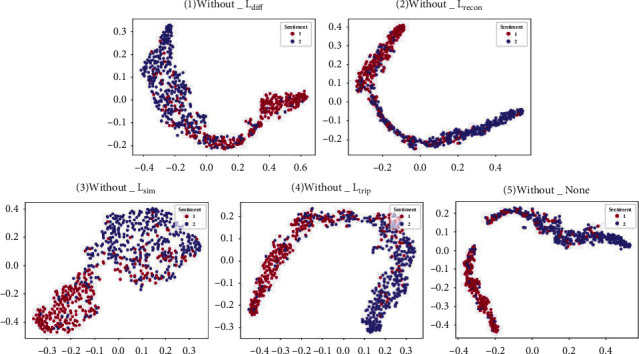
Visualization of sentiment semantic distribution under different loss. Notes: (1) lack of loss function Ldiff; (2) lack of loss function Lrecon; (3) lack of loss function Lsim; (4) lack of loss function Ltrip; (5) full configuration of loss function.

An ablation experiment is designed in this section. The network is retrained after obtaining a zero setting of the loss weights (*α*, *β*, *λ*, *η*) of other components except for the basic task loss L_task_, and the best performance model parameters are saved. Moreover, to intuitively observe the effects of various loss functions on the model results, the fusion representation of MOSI test samples is visualized by T-SNE, as shown in Figure 5.

As shown in Figure 5, the red spots represent positive emotions, and the blue ones represent negative emotions. When the distance between spots of the same color is shorter and the distance between spots of different colors is farther, the effect of semantic clustering and emotion analysis is better. The figure shows the T-SNE graph of the test data fusion representation, showing different distribution features under different loss function training. When all component losses exist, the model has the best semantic clustering effect. When the weight *γ* of the reconstruction loss L_recon_ is zero, it has the suboptimal clustering effect. When similarity loss L_sim_ does not exist, the clustering effect of the model is the most divergent. The impact of the loss L_diff_ and L_trip_ is between similarity loss and reconstruction loss. Furthermore, to explore the effect of each loss more specifically, the evaluation indexes of the best model of each experiment are recorded in Table 5 for quantitative analysis.

#### 5.3.2. Quantitative Analysis

As shown in Table 5, the model achieves the best performance when all losses are involved. This finding indicates that each component loss is effective. The observation results show that the model is sensitive to L_sim_ and L_diff_. It means that decomposing modes into independent space is conducive to the performance improvement of the model. The effect of cosine triplet-margin loss on the model is smaller than L_sim_ and L_diff_. Because semantic clustering effect is observed in the process of modal similarity feature acquisition. Therefore, the effect of this loss is weakened. In addition, the model is less dependent on reconstruction loss. The reason is that the trivial representation features of a specific encoder can be learned by L_task_ in the absence of reconstruction loss. The model is most sensitive to similarity loss; thus, the selection of similarity loss is very important. Therefore, an in-depth analysis is discussed in the following section.

### 5.4. Comparison of Similarity Measures

**Figure 6:**
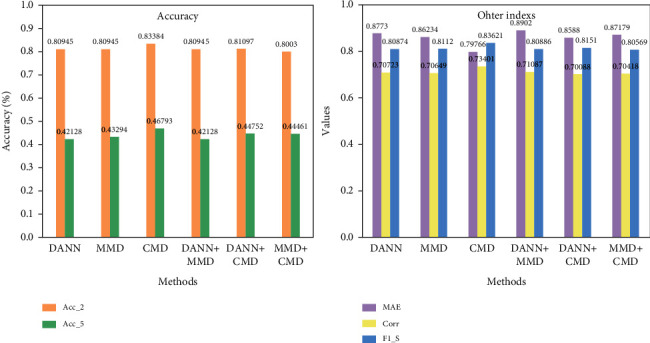
Visualization of performance comparison under different similarity loss.

In this section, the selection of similarity loss function in 3.4.2 is discussed. For this reason, the following experiment is designed. Domain adversarial loss (DANN) [48], maximum mean square measure (MMD) [49], CMD, and their combinations are used for network training tests, as shown in Figure 6. The first three columns in the figure show that the performance of CMD in a single form is better than that of MMD and DANN in various indexes.

The reasons are summarised in the following points: (i) CMD can directly perform exact matching of the high-order moment without expensive distance and kernel matrix calculation; (ii) compared with CMD, DANN obtains modal similarity through minimax game using discriminator and shared encoder. However, in adversarial training, additional parameters are added, and fluctuations may be encountered in training. Moreover, through the observation of joint form (the last three columns), the effect of similarity loss with CMD is better than that of the loss without CMD but worse than that of single CMD loss. This finding indicates that the increase in computation cost reduces the efficiency of network learning and further verifies the rationality of selecting CMD as similarity loss.

## 6. Conclusions

This paper studies multimodal emotion analysis. In the research, we have the following findings: (1) feature representation with more comprehensive information can reduce the burden of fusion network; (2) the redundant information of each mode can be used more effectively by jointing modality-invariance and modality-specificity representations of each mode; (3) simple dynamic fusion mechanism can obtain the interaction between modes more efficiently. Thus, this study puts forward a multimodal sentiment analysis framework consisting of two parts, namely, improved JDSN and HGFN. Firstly, modal invariant-specific joint representation of each mode is obtained through an improved JDSN module to effectively utilize the complementary information amongst modes and reduce the heterogeneity gap between modes. Then, the joint representation of each mode is input to the HGFN for fusion to provide input for the prediction network. Moreover, a new combined loss function is designed to encourage the DISRFN model to learn the representation of expectation. Finally, the performance analysis experiment is carried out on MOSI and MOSEI data sets, obtaining acceptable results. In practice, the multimodal data usually have an unbalanced phenomenon, which will lead to the task bottleneck of the model. However, the study does not consider this issue. Therefore, we plan to study the problems of multimodal imbalance in the future.

## Figures and Tables

**Table 1:** Hyperparameter settings in this article.

Hyperparameter	MOSI	MOSEI
CMD K	5	5
Batch_size	16	16
*α*	0.3	0.4
*β*	1.0	0.8
*γ*	0.4	0.4
*η*	0.1	0.01
Drop	0.4	0.1
Hid	256	256
P_h	64	50

**Table 2:** Comparison experiments of multimodal models in MOSI

Model	MAE	Mul_Acc2	Mul_Acc5	Corr	F1_Score	CPU_Clock
TFN [11]	1.016	0.765	0.386	0.604	0.765	0.404
LMF [12]	1.009	0.767	0.362	0.604	0.769	0.395
MFN [9]	1.007	0.773	0.329	0.632	0.773	0.379
ARGF [15]	0.857	0.814	0.423	0.712	0.815	0.147
Gragh-MFN [10]	1.003	0.784	0.360	0.623	0.785	0.454
MARM [20]	1.028	0.756	0.351	0.625	0.755	0.345
LSTHM [20]-AttFusion	1.087	0.745	0.375	0.608	0.744	1.527
LSTHM [20]-Concat	1.056	0.750	0.370	0.581	0.752	1.524
LSTHM [20]-DFG	0.992	0.758	0.401	0.626	0.757	0.357
LSTHM [20]-Out_Product	1.092	0.764	0.332	0.569	0.764	0.708
MISA [41]	0.827	0.819	0.440	0.726	0.819	0.839
**DISRFN (ours)**	**0.798**	**0.834**	**0.468**	**0.734**	**0.836**	**0.737**

**Table 3:** Comparison experiments of multimodal models in MOSEI.

Model	MAE	Mul_Acc2	Mul_Acc5	Corr	F1_Score	CPU_Clock
TFN [11]	0.714	0.760	0.443	0.507	0.761	0.417
LMF [12]	0.729	0.761	0.436	0.520	0.760	0.412
MFN [9]	0.715	0.773	0.432	0.530	0.772	0.418
Gragh-MFN [10]	0.714	0.765	0.448	0.526	0.766	0.46
MARM [20]	0.708	0.772	0.449	0.530	0.773	0.363
LSTHM [20]-AttFusion	0.852	0.733	0.383	0.403	0.733	1.585
LSTHM [20]-Concat	0.861	0.704	0.383	0.383	0.721	1.6
LSTHM [20]-DFG	0.837	0.748	0.391	0.437	0.748	0.369
LSTHM [20]-Out_Product	0.905	0.722	0.383	0.405	0.723	0.715
MISA [41]	0.600	0.858	0.538	0.776	0.857	0.975
**DISRFN (ours)**	**0.591**	**0.875**	**0.541**	**0.781**	**0.875**	**0.948**

**Table 4:** Experiments of fusion methods.

Method	MAE (↓)	Mul_Acc2 (↑)	Mul_Acc5 (↑)	Corr (↑)	F1_Score (p/g) (↑)
JDSN-AttFusion	0.924	0.791	0.378	0.687	0.782
JDSN-concat	0.839	0.814	0.443	0.724	0.813
JDSN-DFG	0.825	0.816	0.459	0.727	0.817
**DISRFN (ours)**	**0.798**	**0.834**	**0.468**	**0.734**	**0.836**

**Table 5:** Experiments of ablation study.

Method	MAE	Mul_Acc2	Mul_Acc5	Corr	F1_Score
Without diff loss	0.868	0.811	0.404	0.728	0.816
Without sim loss	0.999	0.784	0.351	0.723	0.782
Without recon loss	0.833	0.817	0.464	0.711	0.816
Without CosineTriplet loss	0.857	0.799	0.469	0.705	0.798
**ALL loss**	**0.798**	**0.834**	**0.468**	**0.734**	**0.836**

## Data Availability

The data used includes MOSI and MOSEI. The address of the MOSI dataset is correct. The MOSEI dataset address is as follows: http://immortal.multicomp.cs.cmu.edu/raw_datasets/CMU_MOSEI.zip.
